# Extracts from the Records of the Chicago Academy of Medical Sciences

**Published:** 1860-01

**Authors:** S. C. Blake

**Affiliations:** Sec.


### EXTRACTS FROM THE RECORDS OF THE CHICAGO ACADEMY OF MEDICAL SCIENCES.

(S. C. BLAKE, M. D., SEC.)

August 1st, 1859.

Dr. Miller reported two cases of diphtheria, which disease has been prevailing to some extent in this city. The disease was ushered in by chill and fever; tonsils and fauces edæmatous, and of a livid color, with dirty yellow membranous exudations.

Dr: Macalister reported four cases in his own practice, and stated that there had been three cases of death from the same disease in his neighborhood, treated by an undergraduate.

Dr. H. N. Hurlbut reported three cases which had occurred in his practice.

Dr. Macalister adopted the treatment recommended by the "*London Lancet*" in his cases, and found it to be very successful, viz: sesquichloride of iron and chlorate of potash internally, and the hydrochloric acid as a local application.

Dr. Holmes read the report of a case of spontaneous gangrene which occurred in Dr. Schlœtzer's practice.

Dr. Holmes stated that Dr. Virchrou had found in a number of post mortem examinations, that the arteries just above the diseased part were plugged with fibrinous clots; and it was the opinion of Dr. Virchrou that these clots were formed in some of the larger trunks of the arteries, and perhaps in the heart itself, and were floated down to the bifurcation of the arteries, where they were found, thus arresting the arterial circulation.

Dr. Ingals thought the clots might have been formed in consequence of diseased arteries, the blood becoming arrested in its course, and thereby forming fibrinous clots which finally plugged the vessel.

Dr. Holmes replied that the cases almost always occurred suddenly, and in the cases above referred to as examined by Dr. Virchrou, the arteries surrounding the clots were healthy.

Dr. Macalister related a case which he saw in the Albany Hospital, the patient having suffered from menorrhagia.

Dr. Bloodgood spoke of a case which he once saw, where the gangrene commenced in the toes and extended throughout the entire limb, and therefore he thought could not have been produced in the manner related by Dr. Virchrou.

Dr. Miller thought the theory of the formation of blood clots in the heart might also account for the sudden death of parturient women on raising them towards an erect position soon after severe flooding.

Dr. Bloodgood did not believe the above to be the cause of death in such cases as had been mentioned by Dr. Miller, from the well known fact that many of such patients, after fainting and insensibility had occurred, were restored by placing them again in a horizontal position.

On motion of Dr. Hamill, voted to change the time of holding the regular meetings of the Academy to the first Friday evening of each month.

On motion of Dr. Davis, voted to instruct the secretary to print the fee table.

Oct. 7th. Dr. Holmes reported two very interesting cases of injury to the eye.

Dr. Chas. G. Smith exhibited a very interesting specimen of salivary calculous.

Nov. 4th. The annual address was delivered by Dr. James Bloodgood; and on motion of Dr. Rauch, a copy was requested for publication.

Dr. Fisher reported a case of necrosis of the inferior maxillæ. Dr. Fisher removed a sequestrum, which he exhibited to the Academy.

Dr. Fisher exhibited a very fine specimen of necrosis of the head of the fibula and tibia, and lower extremity of the femur, resulting from bad treatment by an irregular practitioner. Dr. F. amputated the limb.

